# Expanding horizons: new roles for non-canonical RNA-binding proteins in cancer

**DOI:** 10.1016/j.gde.2017.11.006

**Published:** 2018-02

**Authors:** Samantha Moore, Aino I Järvelin, Ilan Davis, Gareth L Bond, Alfredo Castello

**Affiliations:** 1 Department of Biochemistry, University of Oxford, United Kingdom; 2 Ludwig Institute for Cancer Research, Nuffield Department of Medicine, University of Oxford, United Kingdom

## Abstract

Cancer development involves the stepwise accumulation of genetic lesions that overcome the normal regulatory pathways that prevent unconstrained cell division and tissue growth. Identification of the genetic changes that cause cancer has long been the subject of intensive study, leading to the identification of several RNA-binding proteins (RBPs) linked to cancer. Cross-reference of the complement of RBPs recently identified by RNA interactome capture with cancer-associated genes and biological processes led to the identification of a set of 411 proteins with potential implications in cancer biology. These involve a broad spectrum of cellular processes including response to stress, metabolism and cell adhesion. Future studies should aim to understand these proteins and their connection to cancer from an RNA-centred perspective, holding the promise of new mechanistic understanding of cancer formation and novel approaches to diagnosis and treatment.

**Current Opinion in Genetics & Development** 2018, **48**:112–120

This review comes from a themed issue on **Cancer genomics**

Edited by **Fátima Gebauer** and **Omar Abdel-Wahab**

For a complete overview see the Issue and the Editorial

Available online 5th December 2017

**https://doi.org/10.1016/j.gde.2017.11.006**

0959-437X/© 2017 The Authors. Published by Elsevier Ltd. This is an open access article under the CC BY license (http://creativecommons.org/licenses/by/4.0/).

## Introduction

Genetic and epigenetic mutations can lead to dysregulation of the cellular mechanisms controlling cell fate, ultimately causing cancer. The hallmarks of cancer include uncontrolled cell proliferation in the absence of external cues, resistance to cell death, evasion of growth suppressors and the immune system, metabolic reprogramming, tissue invasion and metastasis, and sustained angiogenesis [1]. Identification of causal genetic variants in cancer has long been the subject of intensive study, and a number of these have been mapped to RNA-binding proteins (RBPs). RBPs assemble dynamic complexes with RNA, termed ribonucleoproteins (RNPs), that mediate virtually every stage of the RNA lifecycle [2]. Cancer-conducive mutations and mis-expression of RBPs affect most if not all steps of RNA metabolism, including RNA splicing (e.g. SRSF2) [3], 3′ end processing (e.g. CPEB1) [4, 5], editing (e.g. ADAR1) [6, 7], stability (e.g. ZFP36) [8], storage and localisation (e.g. IMP/IGF2BP proteins) [9], translation (e.g. eIF4E) [10], and biogenesis of small RNAs such as miRNAs (e.g. AGO2, LIN28) [11]. Alterations of RNA metabolism due to RBP dysfunction can cause global changes in the transcriptome and proteome of the cell that can affect cell growth, proliferation, invasion and death. Most of the RBPs studied in the context of cancer are *bona fide*, canonical RBPs characterised by the presence of canonical RNA-binding domains (RBDs) such as RNA-recognition motifs (RRMs) [12]. The functions of such canonical RBPs in cancer aetiology have been recently reviewed elsewhere [13•, 14, 15, 16]. However, studies over the past three decades have identified many RBPs harbouring non-canonical RBDs, whose roles in cancer remain largely unknown. The complement of non-canonical RBPs was largely expanded with the recently developed proteome-wide approaches for unbiased identification of RBPs, uncovering hundreds of proteins lacking classical RBDs endowed with RNA-binding activity [17, 18]. Amongst many other diverse molecular functions, these non-canonical or unorthodox RBPs include cell cycle regulators, metabolic enzymes, protein scaffolds and antiviral factors. In this review, we aim to highlight the emerging roles of non-canonical RBPs in cancer.

### Identifying cancer-linked RBPs

**Figure 1:**
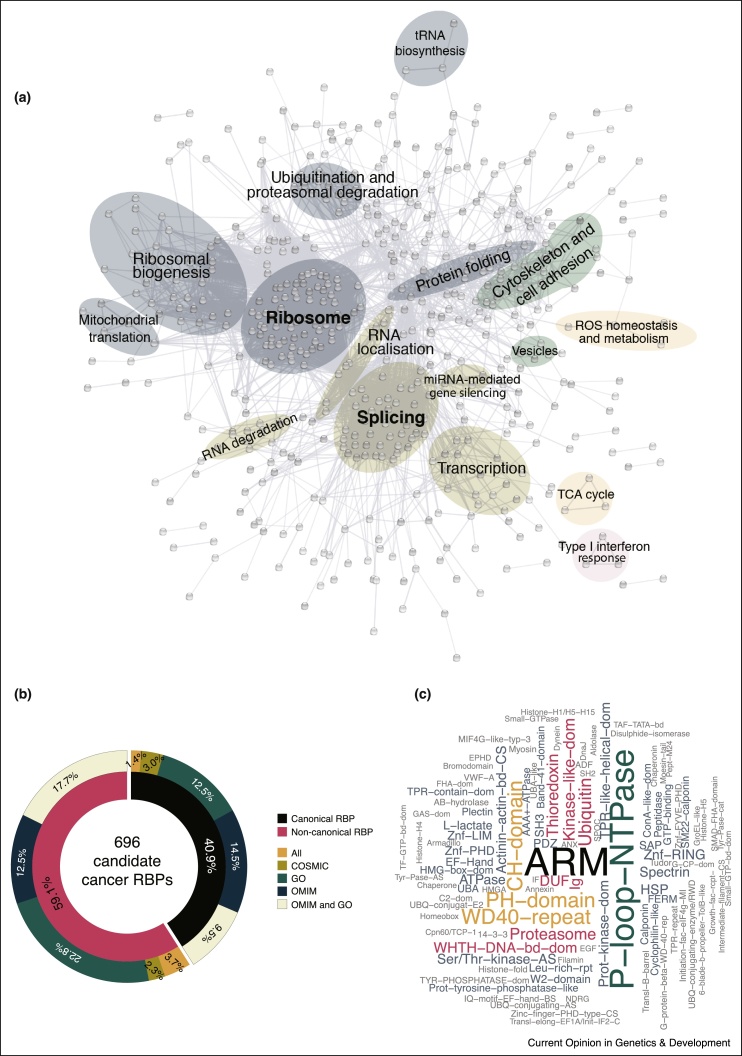
RNA-binding proteins linked to cancer. **(a)** STRING [61] network showing connections between RNA-binding proteins (RBPs) with links to cancer based on annotations in COSMIC, OMIM, and GO (see main text). Main network hubs are highlighted. **(b)** Sunburst graph showing breakdown of cancer-linked RBPs by RBD classification (i.e. classical or non-canonical) and source of cancer-link information. **(c)** Word cloud of domains present in unorthodox cancer-linked RBPs. Size indicates relative prevalence.

**Figure 2:**
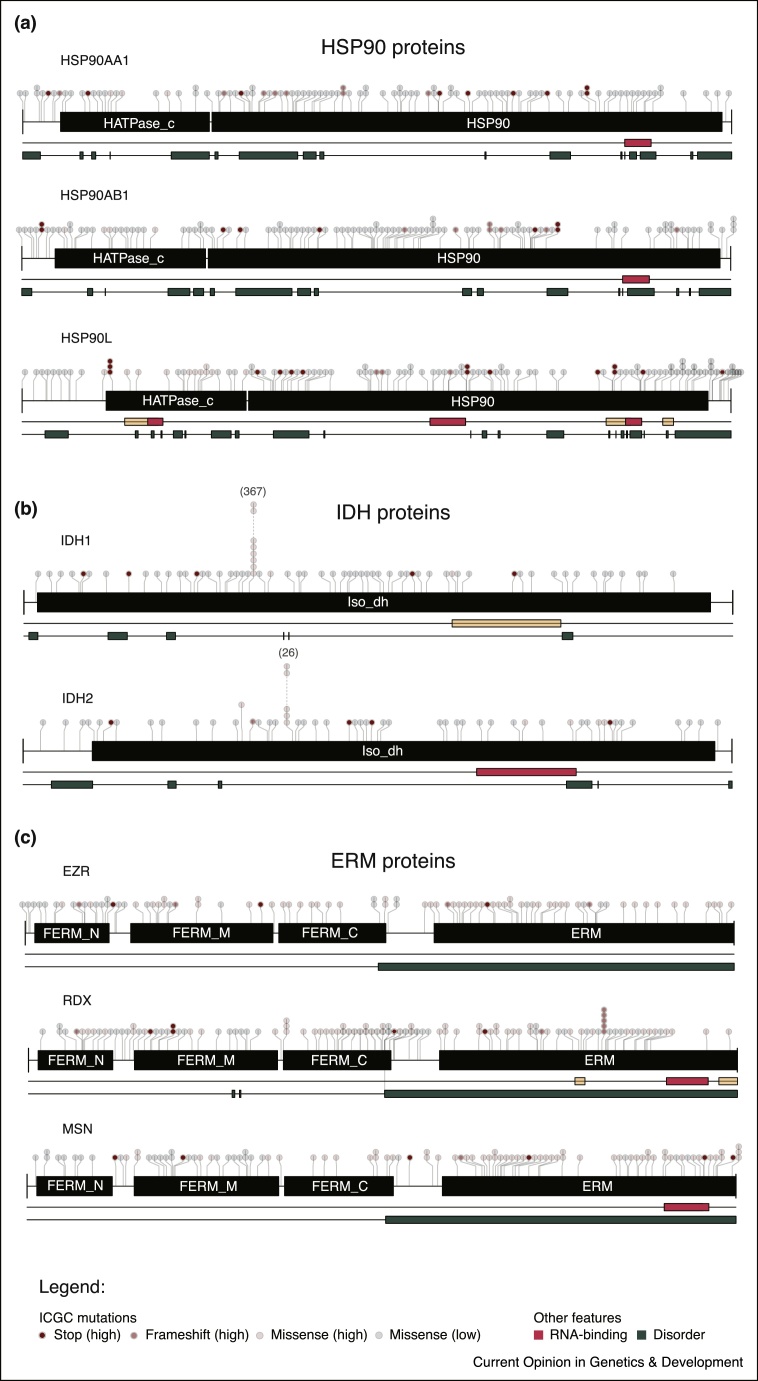
Examples of unorthodox cancer-linked RBPs. Architecture of **(a)** RNA-binding HSP90 proteins **(b)** IDH proteins 1 and 2 **(c)** the three ERM proteins. Lollipops indicate the cancer-associated mutations available in the ICGC data portal [62], black boxes represent Pfam-annotated [63] protein domains, red and orange boxes map the high-confidence and candidate RNA-binding sites reported by RBDmap [20^••^], respectively, and green boxes indicate regions which are predicted to be intrinsically disordered (IUPred score > 0.4) [64]. Frequently mutated residues in IDH1 and IDH2 proteins with established links to several cancers (see text for further information), are denoted by the number of reported mutations in parentheses.

To systematically determine the complement of RBPs with potential roles in cancer, we compared the census of human RBPs collected in system-wide studies by RNA interactome capture (RNA-IC) [19^•^] and RBDmap [20^••^] with cancer-associated proteins annotated in the Catalogue of Somatic Mutations in Cancer (COSMIC) cancer gene census list [21], Online Mendelian Inheritance in Man (OMIM) [22], or related processes in Gene Ontology (GO) [23]. RNA-IC employs ultraviolet crosslinking, oligo (dT) capture and mass spectrometry to identify RBPs [17, 18]; RBDmap extends this protocol, utilising a controlled proteolytic step to determine the regions within RBPs engaged in the interaction with RNA [20^••^]. Comparison of the RNA-bound proteome with cancer proteins resulted in the identification of 696 RBPs with potential implications in cancer biology (Figure 1, Table 1 and Supplementary Table 1). Seventy-three of these RBPs were present in COSMIC, 435 in OMIM, and 477 were annotated by a GO term related to cancer. The fact that half the RNA-bound proteome is related here to cancer highlights the importance of RBPs in cell-fate decisions. Splicing and translation emerge as predominant processes for cancer-related RBPs (Figure 1a). Additionally, functions commonly associated with cancer, including metabolic remodelling, cell adhesion, and interferon response, are also observed (Figure 1a). Strikingly, the set of cancer-related RBPs includes a similar proportion of proteins harbouring well-established RBDs (40.9%) and proteins lacking a recognisable RBD (59.1%) (Figure 1b). Protein-protein interaction domains such as ARM and WD40, and enzymatic cores such as P-loop-NTPase, kinase-like and thioredoxin domains, emerged as predominant signatures amongst unorthodox, cancer-related RBPs (Figure 1c). Highlighting the links of unorthodox RBPs to cancer, numerous cancer-related mutations were identified throughout the sequence of these proteins, including missense mutations, frameshifts and premature stop codons (Figure 2 and Supplementary Figures 1–3). Some of these mutations overlap with proteins’ non-canonical RBDs (uncovered by RBDmap [20^••^]), invoking potential effects on RNA binding. Below, we have selected a few families of these non-canonical cancer-linked RBPs for further discussion.

**Table 1:** Selected unorthodox cancer-linked RBPs

Gene symbol	Function(s)	RBDmap	Cancer resources	Cancers
CDKN2A	Alternative transcripts. Cell cycle arrest and tumour suppressor functions.	−	COSMIC, OMIM, GO	Melanoma, pancreatic and multiple cancers, familial malignant melanoma
CLTC	Component of clathrin-coated vesicles and pits, involved in intracellular vesicle transport.	−	COSMIC, OMIM, GO	ALCL, renal
IDH1	Cytosolic and peroxisomal NADP-dependent isocitrate dehydrogenase.	−	COSMIC, OMIM, GO	Glioblastoma
EIF3E	Subunit of eIF3 complex. Role in translation initiation.	−	COSMIC, OMIM, GO	Colorectal
JUN	Component of API-1 complex transcription factor.	−	COSMIC, OMIM, GO	Sarcoma
MYH9	Heavy chain component of nonmuscle myosin II. Actin cytoskeleton binding and remodelling.	−	COSMIC, OMIM, GO	ALCL
ATP1A1	Catalytic subunit of Na(+), K(+) ATPase. Generates plasma membrane electrochemical gradient.	+	COSMIC, OMIM, GO	Adrenal aldosterone producing adenoma
NPM1	Nucleolar-nuclear-cytoplasmic shuttling protein. Multiple functions including histone and protein chaperone.	+	COSMIC, OMIM, GO	NHL, APL, AML
POU5F1	POUhomeodomain-containing transcription factor. Role in embryonic development and stem cell pluripotency.	−	COSMIC, OMIM, GO	Sarcoma
RPL5	Ribosomal protein component of 60S ribosome. Binds 5S rRNA to form 5S RNP, transports to nucleolus for ribosomal assembly.	+	COSMIC, OMIM, GO	T-ALL
TFRC	Cell surface receptor mediating iron uptake via receptor-mediated endocytosis.	−	COSMIC, OMIM, GO	NHL
TPR	Structural component of nuclear pore complex, crucial for nucleocytoplasmic transport.	−	COSMIC, OMIM, GO	Papillary thyroid, NSCLC
EZR	Intermediate linking plasma membrane and cytoplasm. Key for cell adhesion, migration, structural stability and plasma membrane structures.	−	COSMIC, OMIM, GO	NSCLC
YWHAE	Adapter protein. Role in signal transduction/signalling pathways, by binding multiple protein partners.	−	COSMIC, OMIM, GO	Endometrial stromal sarcoma
DEK	Role in chromatin organisation and splice site selection during pre-mRNA processing.	−	COSMIC, OMIM, GO	AML
CALR	Major calcium binding storage protein and molecular chaperone in ER lumen	−	COSMIC, OMIM, GO	Myeloproliferative neoplasms, myelodysplastic syndromes
HIST1H4I	Involved in nucleosome formation and organisation.	+	COSMIC, OMIM, GO	NHL
PSIP1	Transcriptional coactivator.	−	COSMIC, OMIM	AML
SF3B1	Mediates formation and anchoring of U2 snRNP to pre-mRNA upstream of intronic branch sites.	+	COSMIC, OMIM	Myelodysplastic syndromes
HLA-A	Member of the HLA class I heavy chain paralogues. Presents peptides derived from ER lumen to the immune system.	−	COSMIC, OMIM	Spitzoid tumour
MYO5A	Myosin 5 heavy chain member. Actin-based motor protein involved in spindle pole assembly and vesicle/mRNA transport.	−	COSMIC, OMIM	Spitzoid tumour
KMT2C	Histone methyltransferase, specifcally methylating H3 Lys-4 (epigenetic mark associated with transcriptional activation)	−	COSMIC, OMIM	Medulloblastoma
TOP1	Alters DNA topology during transcription and replication. Transiently cleaves and rejoins DNA strands.	−	COSMIC, OMIM	AML
PHF6	Proposed role in transcription and/or chromatin remodelling.	−	COSMIC, OMIM	ETP ALL
APOBEC3B	Cytidine deaminase with role in retrovirus replication and retrotransposon movement inhibition.	−	COSMIC, OMIM	Breast cancer
THRAP3	Component of spliceosome, involved in pre-mRNA splicing and mRNA decay. Potential role as transcriptional coactivator.	−	COSMIC, OMIM	Aneurysmal bone cyst
SND1	Transcriptional coactivator. Part of the RISC complex. Roles in RNA editing, mRNA splicing and stability.	+	COSMIC, GO	Pancreas acinar carcinoma
KTN1	Integral ER protein. Binds kinesin, suggesting role in intracellular vesicle/organelle mobility.	−	COSMIC, GO	Papillary thyroid

Candidate unorthodox RBPs that are present in at least two cancer resources (COSMIC cancer gene census, OMIM and GO), and are identified as RBPs in more than two independent datasets (RBDmap or RNA-IC), are listed here. The complete list, together with additional analyses are available in Supplementary Table 1 (ALCL = anaplastic large cell leukaemia, NHL = non-Hodgkin lymphoma, APL = acute promyelocytic leukaemia, AML = acute myelogenous leukaemia, T-ALL = T-cell acute lymphoblastic leukaemia, NSCLC = non-small cell lung cancer, ETP ALL = early T-cell precursor acute lymphoblastic leukaemia).

### Chaperones and protein scaffolds

Heat shock proteins (HSPs) are highly conserved molecular chaperones that play important roles in protein synthesis, localisation and degradation, whilst preventing the accumulation of potentially pathogenic protein aggregates. HSPs confer key cytoprotective effects to cells under physiological stresses, facilitating cell survival. HSP overexpression has been observed in a range of cancers, where their cytoprotective and anti-apoptotic effects are exploited to aid tumour growth, disease progression and metastasis [24]. HSP substrates include proteins involved in key oncogenic signalling pathways. Accordingly, the utility of HSP inhibitors in cancer therapy is under extensive investigation, due in part to their ability to target multiple proteins and upstream components of tumour-associated pathways [25].

Several HSPs have been consistently classified as RBPs by RNA-IC and RBDmap, including HSP90, HSP70, HSP60, HSP40 and HSP27 protein family members [19•, 20••] (Supplementary Table 1 and Supplementary Figure 1). For example, HSP90aa1 is classified as canonical RBP here due to the presence of the Ribosomal S5 D2-type fold (residues 292–547). However, RBDmap assigns the RNA-binding activity to the non-canonical HSP90 domain and this is consistent across protein homologues (Figure 2a). HSP90aa1 is upregulated in diffuse large B cell lymphoma (DLBCL), along with the key oncoprotein and transcriptional repressor BCL6. HSP90aa1 upregulates both mRNA and protein levels of BCL6 *in vitro*, and HSP90 inhibition can induce antitumour effects in BCL6 positive DLBCL mouse models [26].

In addition to HSP90, RBDmap identified high confidence RNA-binding sites within the heat shock domains of HSP70 and HSP27, which are conserved across homologous proteins [20^••^]. This conservation suggests that RNA-binding activity is a shared property among HSPs. Strikingly, *in vitro* experiments show that certain HSPs can bind AU-rich elements (ARE) in the 3′-UTR of their target mRNAs, mediating RNA decay [27]. RNA binding appears to be independent of chaperone and ATPase activities, at least for HSP70 [28]. HSPs play key roles in RNA metabolism, including miRNA loading in the RNA-induced silencing complex (RISC) [29] and folding of nascent polypeptides concomitantly to ribosome's activity [30]. miRNA and ribosomal activities are central in the regulation of the cellular proteome, invoking potential implications of RNA metabolism in the function of HSPs in cancer development.

Protein–protein interaction domains have been identified as novel RNA-binding sites in unorthodox RBPs [20^••^]. This is the case in four out of seven members of YWHA protein family, namely YWHAG, YWHAE, YWHAB and YWHAZ [19•, 20••], all of which have described links with cancer or related pathways in the literature [31, 32] (Supplementary Table 1 and Supplementary Figure 2). YWHA proteins bind serine/threonine phosphorylation motifs in target proteins, thus regulating their activity, localisation, post-translational modification status and interactions. Recent studies proposed that YWHA family harbours chaperone-like activities, preventing protein aggregation, assisting HSPs in protein refolding, and clearing misfolded proteins [33]. YWHA proteins are therefore able to regulate a vast number of proteins involved in key cellular processes, with implications for tumorigenesis and cancer progression, such as cell cycle progression and arrest, [34], apoptosis [35] and EMT [36], along with key oncoproteins, such as p53 [37], and components of oncogenic pathways [31, 38, 39]. YWHAB, YWHAG and YWHAZ harbour a conserved RBD within the 14-3-3 domain [20^••^] overlapping with partially disordered regions. Owing to the highly conserved nature of these proteins, it is expected that YWHAE would bind RNA as well, and indeed RNA binding is seen in the YWHAE 14-3-3 domain in mouse (Supplementary Table 1) [40]. The 14-3-3 domain consists of several alpha helices, which form the amphipathic protein-interacting groove, and include regions of charged and polar amino acids (helices 3 and 5) and hydrophobic amino acids (helices 7 and 9) [41], which provide suitable potential RNA-binding interfaces. The role of RNA binding in YWHA family function and its implications in cancer development deserve further study.

### Metabolic enzymes

Metabolic reprogramming is an emerging hallmark of cancer [1], required to meet the increased energetic and biosynthetic demands of rapidly proliferating and dividing tumour cells. The RNA-bound proteome is populated by many metabolic enzymes, which reinforces and expands previous *in vitro* and *in vivo* data showing that several enzymes of the intermediate metabolism ‘moonlight’ as RBPs and provide a new regulatory layer between metabolism and gene expression [42, 43]. For example, the isocitrate dehydrogenase enzymes IDH1 and IDH2 function as RBPs [19•, 20••], and are strongly linked to cancer. IDH enzymes catalyse the reversible conversion of isocitrate and NADP+ to α-ketoglutarate (a-KG) and NADPH, a key process in the tricarboxylic acid (TCA) cycle, lipid biosynthesis and NADPH production. IDH2 binds RNA via the dehydrogenase domain, as has been observed with other metabolic enzymes [20••, 43] (Figure 2b). This region is also conserved in IDH1, and indeed shows RNA binding activity in mouse (Supplementary Table 1) [40]. How IDH1/2 RNA-binding activity influences its biological activity is not known. However, mechanisms determined for other moonlighting enzymes offer some possible clues.

Aconitase 1 (ACO1, also known as iron regulatory protein 1 (IRP1)) assembles with an iron–sulphur [Fe–S] cluster under normal iron levels, forming the catalytically active protein. However, under low iron concentrations [Fe–S] is no longer available, causing ACO1 to remain as an apoprotein. In this state, ACO1 binds the 5′ and 3′ UTRs of mRNAs involved in iron homeostasis and regulates their fate [42]. RNA binding and catalytic activity are mutually exclusive functions that are actually linked to different protein conformations [44]. Another RNA-binding metabolic enzyme is thymidylate synthase (TYMS), a key target of cancer therapy that binds its own RNA in the absence of its substrate. This unusual binding of the enzyme to its mRNA inhibits its translation initiation [45] thus providing a negative feedback loop that regulates the level of the enzyme. TYMS also binds the RNAs of the key tumour suppressor p53 and the key oncogene c-myc [46, 47], suggesting additional possible regulatory roles in cancer development. Substrate (or cofactor)-dependent regulation of protein levels (TYMS) or activity (ACO1) offers a high degree of functional plasticity, allowing rapid responses to alterations in the metabolic state of the cell. Whether IDH1/2 follows a similar strategy to ACO1 or TYMS, and whether it plays key roles in cancer development will be important to explore.

IDH enzymes have well established roles in glioma, secondary glioblastoma and acute myeloid leukaemia (AML). The mechanistic role of IDH in these cancers has been determined by analysing point mutations at specific arginine residues (residues R132 and R172/R140 in IDH1 and IDH2, respectively — Figure 2b) that disrupt catalytic activity, resulting in neomorphic enzymatic activity, and the subsequent accumulation of 2-hydroxyglutarate (D-2HG) from the reduction of a-KG. This metabolic imbalance causes hypermethylation at CG-rich DNA sequences (CpG islands), as D-2HG is able to competitively inhibit a-KG dependent dioxygenases, such as DNA and histone demethylases. These epigenetic lesions result in significant alterations of gene expression [48, 49, 50]. Other lines of evidence have linked this neomorphic enzymatic activity to reduction in NADPH levels, which in turn leads to increased susceptibility to reactive oxygen and nitrogen species, due to lack of reduced antioxidant enzymes. This renders cells vulnerable to ROS and oxidative DNA damage, which can result in further accumulation of cancer-promoting genetic lesions [51]. However, not all IDH mutations observed in cancer impact catalytic activity, with other observed IDH mutations (Figure 2b) resulting in loss of function, overexpression or having no impact on WT catalytic activity [49, 52]. Knowing that TYMS auto-regulates its protein levels through interaction with its own RNA [45], it is a sensible hypothesis that IDH may follow a similar strategy, and that dysregulation of its RNA-binding activity may result in altered IDH levels. This possibility should be explored in the future.

In addition to IDH1/2, ACO1, and TYMS, thioredoxin-domain containing antioxidant enzymes thioredoxin (TXN) and glutaredoxin 3 (GLRX3), along with catalase (CAT), peroxiredoxin 1 and 3 (PRDX1/PRDX3), were identified as candidate cancer-associated RBPs (Supplementary Figure 3), providing a potentially interesting link between antioxidant metabolism, RNA binding and cancer.

### ERM proteins

All three members of the ezrin-radixin-moesin (ERM) protein family were identified as RBPs by RNA-IC [19•, 20••] (Figure 2c and Supplementary Table 1). The ERM proteins mediate interactions between the actin cytoskeleton and plasma membrane, playing a key role in the organisation of specialised membrane structures, adhesion sites and cell junctions. Additionally, the ERM proteins facilitate signal transduction between intracellular and extracellular compartments, with roles in adhesion and migration [53]. The ERM proteins are suggested to influence cancer progression, including invasion, EMT and metastasis, due to these functions in cell adhesion and migration. Mislocalisation or altered ERM expression can also influence receptor complex formation, thus impacting signal transduction, including key oncogenic pathways such as PI3K/Akt and Wnt/β-catenin pathways [54]. RBDmap in human cells identified RNA-binding activity in a disordered region within the ERM domain in moesin and radixin [20^••^]. Furthermore, the same protein regions in the three ERM proteins were reported by RBDmap to bind RNA in mouse, suggesting conservation of this function across mammals [40]. Interestingly, regulation of ERM proteins by a piRNA-like species has been observed in non-small cell lung cancer lines, but not in normal lung bronchial epithelial lines [55]. This highlights the potential role of RNA in the regulation of protein's function in pathological conditions. The role of RNA-ERM interactions in cytoskeletal configuration, cell adhesion and migration should be studied in the future in the context of cancer development.

## Outlook

Recently developed global approaches for identification of RBPs have uncovered a new universe of RNA-binding activities, many of which have been linked to tumorigenesis, cancer progression, invasion and metastasis. We have provided here an overview of the diversity of non-canonical RBPs and their potential links with oncogenesis. However, the precise roles of most of these unorthodox RBPs in cancer, and the relevance of their RNA-binding activities, remain to be elucidated. In this review, we have limited our discussion to human RBPs that have been experimentally identified by RNA-IC and RBDmap. However, the task of uncovering the cancer-related RNA-binding proteome is by no means complete. Other *in silico*, *in vitro* and *in vivo* approaches for identification of RBPs and RBDs have recently been developed 7[17, 18, 20••, 56, 57, 58•, 59•, 60], with advantages and disadvantages over RNA-IC and RBDmap. For example, while RNA-IC and RBDmap offer high specificity in the identification of RBPs, they are limited to polyadenylated RNA. Some of the alternative approaches do not rely on oligo(dT) capture, opening the possibility to identify RBPs with specificity for non-polyadenylated RNA. In the future, the combination of different RNA-binding and cancer mutation data sources will help expand our knowledge of RBPs and their links to cancer. There is certainly interesting future work to elucidate the roles of novel and non-canonical RBPs in the development and progression of cancer. In the longer term, studying the RNA biology of non-canonical mRNA binding proteins is likely to lead to new avenues for treatments of specific cancers.

## Conflict of interest statement

Nothing declared.

## References and recommended reading

Papers of particular interest, published within the period of review, have been highlighted as:

• of special interest
•• of outstanding interest

## Appendix A Supplementary data

Supplementary Figure 1 Examples of additional heat shock proteins (HSPs) identified as candidate cancer-linked RBPs. Lollipops indicate the cancer-associated mutations available in the ICGC data portal [62], black boxes represent Pfam-annotated [63] protein domains, red and orange boxes map the high-confidence and candidate RNA-binding sites reported by RBDmap [20^••^], respectively, and green boxes indicate regions which are predicted to be intrinsically disordered (IUPred score > 0.4) [64].

Supplementary Figure 2 Examples of additional YWHA domain proteins identified as candidate cancer-linked RBPs. Labels are as in Supplementary Figure 1.

Supplementary Figure 3 Examples of additional metabolic enzymes identified as candidate cancer-linked RBPs. Labels are as in Supplementary Figure 1.

Supplementary Table 1 Cancer-linked, unorthodox RBPs. This table shows the intersection between RNA-binding proteins and cancer resource information. It also shows additional information and analyses as indicated in each individual spreadsheet.

Supplementary File 1 Description of methods relating to the identification of candidate cancer-linked RBPs, Figure 1, Figure 2.
