# Supplementary material for: Expanding horizons: new roles for non-canonical RNA-binding proteins in cancer
Source: Curr Opin Genet Dev. 2018 Feb;48:112–20. doi: 10.1016/j.gde.2017.11.006 (PMC5894799; doi:10.1016/j.gde.2017.11.006)

Supplementary Figure 1

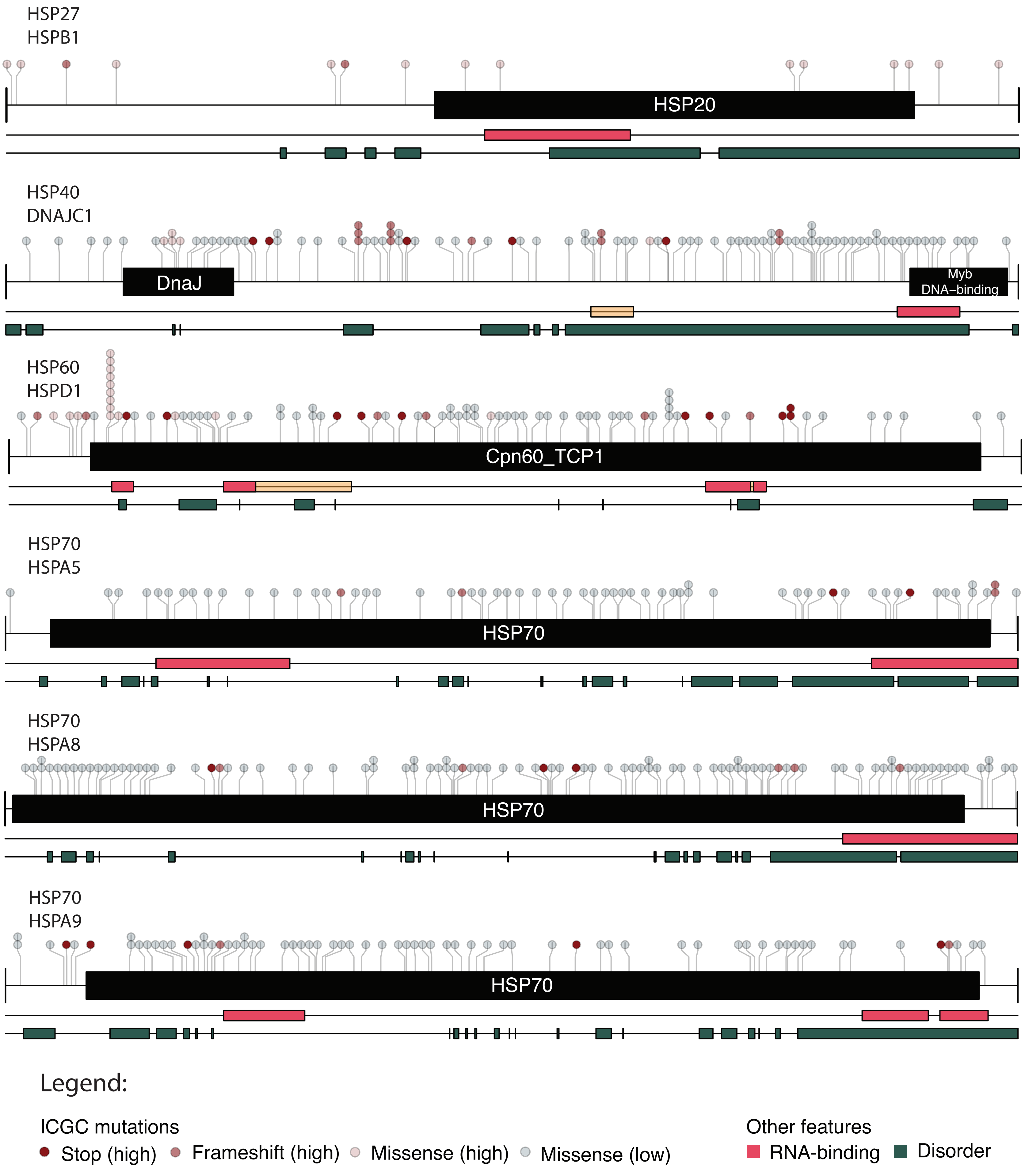

Supplement: Supplementary Figure 1 — Examples of additional heat shock proteins (HSPs) identified as candidate cancer-linked RBPs. Lollipops indicate the cancer-associated mutations available in the ICGC data portal [62], black boxes represent Pfam-annotated [63] protein domains, red and orange boxes map the high-confidence and candidate RNA-binding sites reported by RBDmap [20••], respectively, and green boxes indicate regions which are predicted to be intrinsically disordered (IUPred score > 0.4) [64]. [file mmc1.pdf]
