# Supplementary material for: Expanding horizons: new roles for non-canonical RNA-binding proteins in cancer
Source: Curr Opin Genet Dev. 2018 Feb;48:112–20. doi: 10.1016/j.gde.2017.11.006 (PMC5894799; doi:10.1016/j.gde.2017.11.006)
Supplement: Supplementary Figure 2 — Examples of additional YWHA domain proteins identified as candidate cancer-linked RBPs. Labels are as in Supplementary Figure 1. [file mmc2.pdf]

Supplementary Figure 2

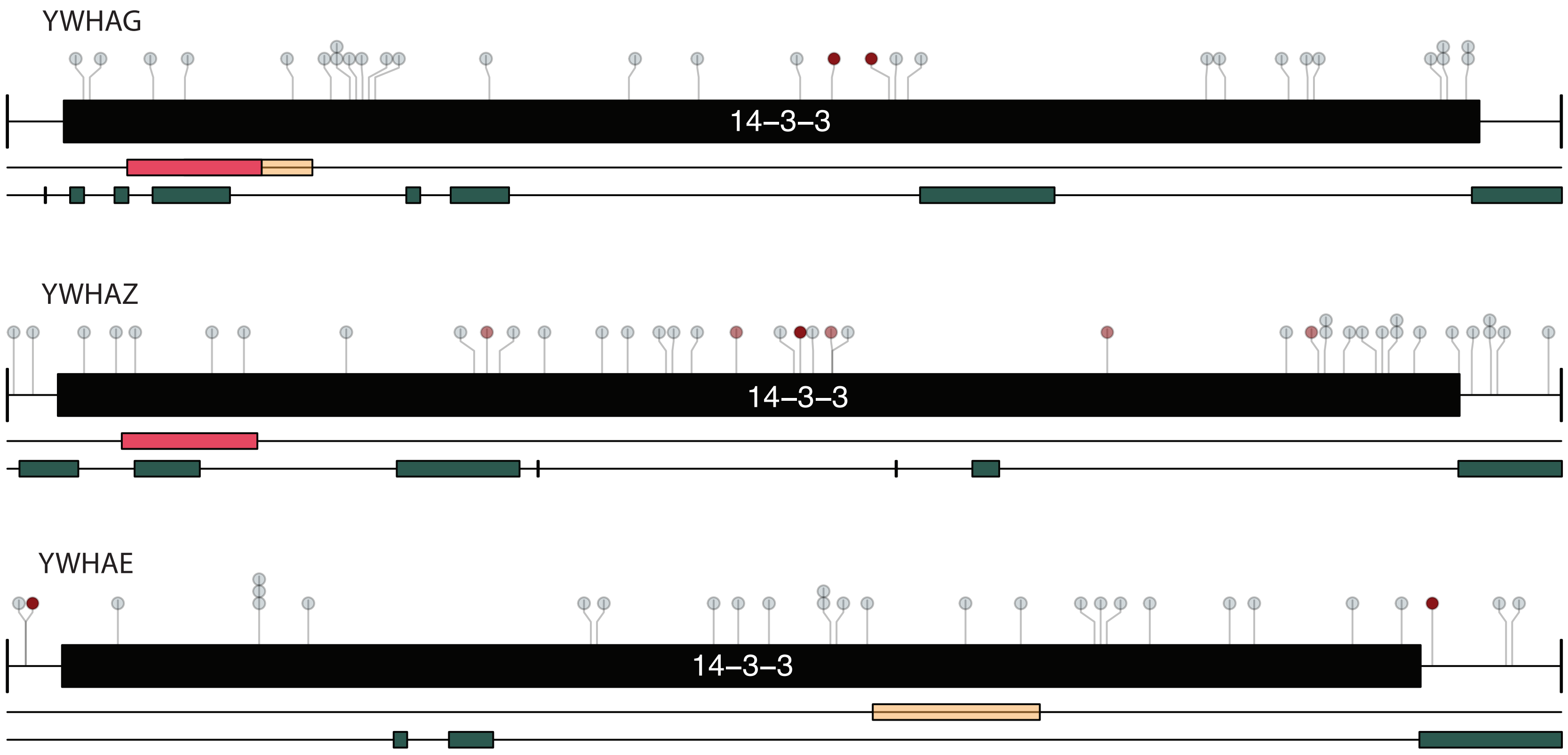

Legend:

ICGC mutations

Stop (high) Frameshift (high) Missense (high) Missense (low)

Other features

RNA-binding Disorder
