# Supplementary material for: Expanding horizons: new roles for non-canonical RNA-binding proteins in cancer
Source: Curr Opin Genet Dev. 2018 Feb;48:112–20. doi: 10.1016/j.gde.2017.11.006 (PMC5894799; doi:10.1016/j.gde.2017.11.006)

Supplementary Figure 3

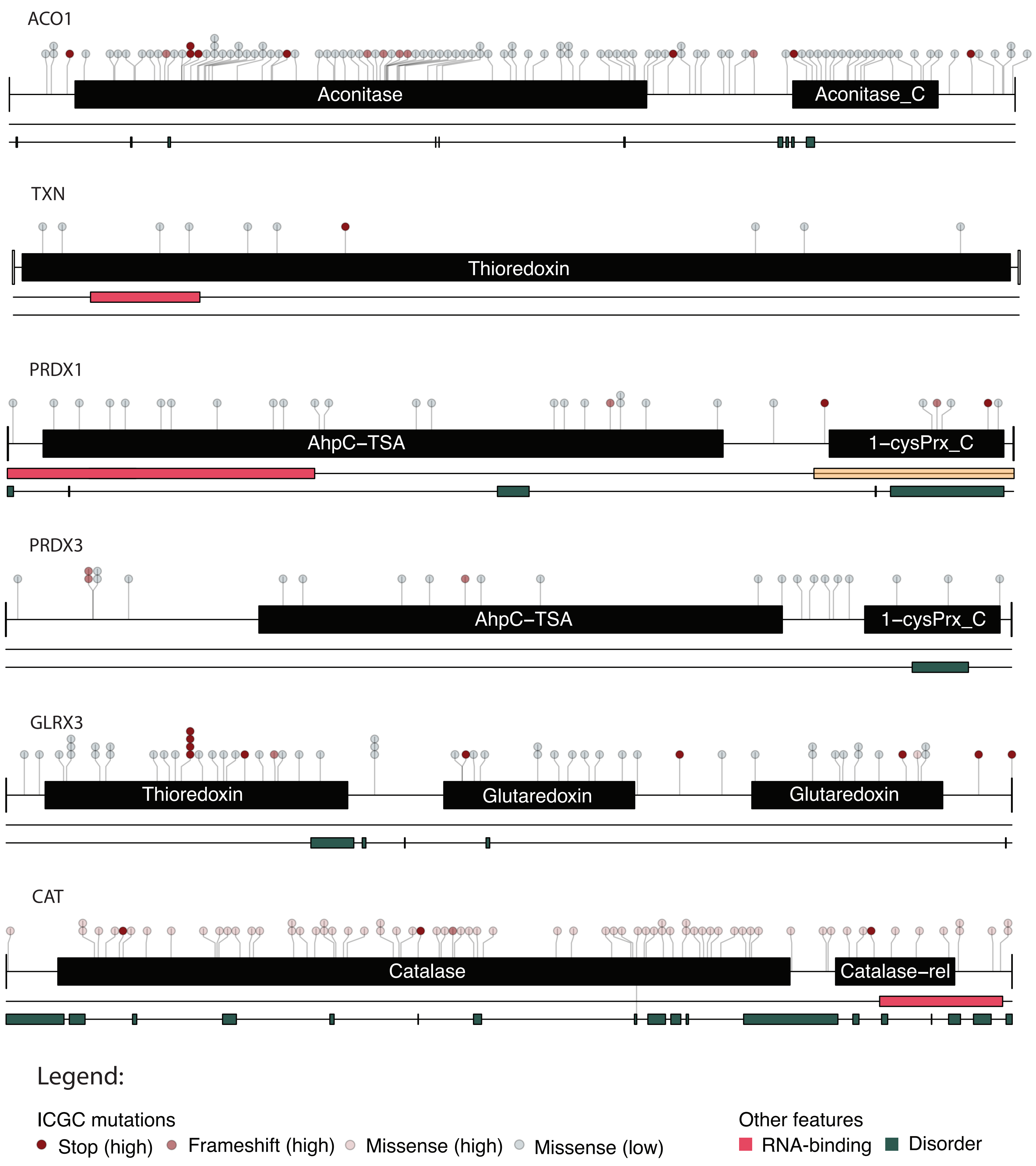

Supplement: Supplementary Figure 3 — Examples of additional metabolic enzymes identified as candidate cancer-linked RBPs. Labels are as in Supplementary Figure 1. [file mmc3.pdf]
