# Supplementary material for: Expanding horizons: new roles for non-canonical RNA-binding proteins in cancer
Source: Curr Opin Genet Dev. 2018 Feb;48:112–20. doi: 10.1016/j.gde.2017.11.006 (PMC5894799; doi:10.1016/j.gde.2017.11.006)
Supplement: Supplementary File 1 — Description of methods relating to the identification of candidate cancer-linked RBPs, Figure 1, Figure 2. [file mmc5.docx]

**SUPPLEMENTARY METHODS**

**Implementation**

All queries, comparisons, and plotting were done using R (https://www.R-project.org).

**Selection of RNA-binding proteins**

For our analyses of RNA-binding proteins (RBPs), we chose the compiled list of proteins identified in human RNA-IC studies from Beckman et al. [1]. Additionally, we included protein identified as RNA-binders in RBDmap [2]. Mapping between different database IDs (Entrez, ENSEMBL, Uniprot, GO, and OMIM) was done using R/Bioconductor package “AnnotationHub” [3].

**Selection of cancer-linked proteins**

We downloaded the list of consensus 616 cancer genes from COSMIC [4]. The genes in this list were compared with the list of RBPs through the reported Entrez IDs. COSMIC hits for each of the candidate cancer RBPs, are listed in Supplementary Table 1 sheet “COSMIC info”. An additional summary of relationship between COSMIC tumour types, mutation types, and RBPs is available in Supplementary Table 1 sheets “COSMIC tumours in RBP” and “COSMIC mutations in RBPs”.

We queried the Online Inheritance in Man (OMIM) database [5] for entries linked to “cancer”, “tumour”, and a number of more specific cancer terms, including “adenocarcinoma”, “leukemia”, and “melanoma”. Full list of search terms is presented in Supplementary Table 1 sheet “OMIM search terms” for query terms. We used the R packages “httr” [6] to query the OMI API (https://omim.org/api) and “xml2” [7] to process the retrieved xml files that included MIM IDs for the searched terms. These were then compared with the MIM identifiers of the RBP list. The matched OMIM terms for each of the candidate cancer RBPs, are listed in Supplementary Table 1 sheet “OMIM info”. An additional summary of relationship between each OMIM search term and candidate cancer-linked RBPs is available in Supplementary Table 1 sheet “OMIM cancer terms in RBPs”.

We compared the list of RBPs to Gene Ontology (GO) [8] annotated biological processes, molecular functions, and cellular compartments closely linked with cancer. Selected GO terms included “DNA repair”, “p53 binding”, and “apoptosome”. Full list of the selected GO terms is provided in Supplementary table 1 sheet “GO search terms”. GO IDs of the selected RBPs were then compared to this list of IDs, limiting our analysis to evidence codes 'EXP', 'IC', 'IDA', 'IMP', 'IPI', 'ISS', 'TAS', and 'IEA'. We used the R/Bioconductor package “GO.db” [9] to map GO IDs of the selected RNA-binding proteins to GO terms. GO terms for each of the candidate cancer RBPs, including separate columns for cancer-linked terms, are listed in Supplementary Table 1 sheet “GO info”. An additional summary of relationship between each term and RBPs is available in Supplementary Table 1 sheet “GO cancer terms in RBPs”.

**Candidate cancer RBPs**

To formulate a list of candidate cancer-linked RBPs, we made an intersection between the proteins in above lists of RNA-binding and cancer resources. Supplementary table 1 sheet “cancerRBPs” tabulates the result of this analysis for candidate cancer-related RBPs (total of 696 proteins). The table is sorted by level of evidence linking the protein to cancer, where we consider the strength of evidence based on type (COSMIC > OMIM > GO) and number of resource the protein is identified in. The additional sheet “cancerRBPs with STUDY info” further lists the human RNA-IC and RBDmap studies where the protein was identified as RNA-binding. Sheet “Non-cancer RBPs” lists those RBPs that did not have an entry in the cancer resources.

**Canonical versus non-canonical RBPs**

Information for each RBP was downloaded in an automated fashion from Uniprot and Interpro domain information extracted from the downloaded xml files using the R package “xml2” [7]. RBPs were classified as canonical or non-canonical based on whether they included a canonical RNA-binding domain in InterPro [10] domain annotation or not. Domains we considered canonical include RRMs, KH domains, dsRBDs, a number of zinc finger domains, but also ribosomal domains. The full list of Interpro domains we classified as canonical can be found in Supplementary Table 1 sheet “Canonical RBDs (Interpro)”. An additional summary of relationship between each domain and candidate cancer-linked RBPs is available in Supplementary Table 1 sheet “Domains in cancer RBPs”.

**Analysis and visualisation presented in Figure 1**

We used the online STRING [11] network analysis tool to visualise the relationships between the 696 candidate cancer-related RBPs. We only considered high confidence (0.700) connections with evidence in experiments or databases, and omitted from the visualisation proteins without any connections. We further coloured this plot to annotated/indicate different types of biological pathways (protein production and degradation, transcription and splicing, metabolism, immune response, and cell skeleton and transport).

We visualised the grouping of the 696 candidate cancer-related RBPs into canonical and non-canonical RBPs and the resource of cancer information in a sunburst plot using the R package “sunburstR” [12].

The relative occurrence of Interpro-annotated protein domains in candidate cancer-linked RBPs that were classified as non-canonical RNA-binders was visualised in a word cloud using the R package “wordcloud” [13]. Only the top 104 domains (present in at least three proteins). For these proteins, near-identical protein domain names were grouped under one umbrella term. The grouping is provided in Supplementary Table 1, sheet “Interpro grouping”.

**Analysis and visualisation presented in Figure 2**

For each selected protein (specifically, Uniprot ID), we downloaded the sequence from Uniprot [14], domains from Pfam [15], and experimentally defined RNA-binding regions from RBDmap [2]. We confirmed that the sequence each each of these resources were identical and calculated predicted disorder score using IUPred [16]. Any amino acid with disorder score > 0.4 was considered disordered.

Cancer mutation information was queried and parsed from the International Cancer Genome Consortium Data Portal (ICGC) [17] API (http://docs.icgc.org/portal/api-endpoints/) using R packages “httr”[6] and “jsonlite” [18]. We checked that the position and amino acid of mutations reported in ICGC matched the sequence obtained from Uniprot. For plotting, we only considered mutations classified as high or low impact were considered in the ICGC portal (i.e. unknowns were omitted). Number of circles indicates how many donors are affected by this mutation. The ‘lolliplot’ visualisation of Figure 2 was done using the R package “trackViewer” [19].

**REFERENCES**

1. Beckmann BM, Horos R, Fischer B, Castello A, Eichelbaum K, Alleaume A-M, Schwarzl T, Curk T, Foehr S, Huber W, et al.: **The RNA-binding proteomes from yeast to man harbour conserved enigmRBPs.** *Nat Commun* 2015, **6**:10127.

2. Castello A, Fischer B, Frese CK, Horos R, Alleaume A-M, Foehr S, Curk T, Krijgsveld J, Hentze MW: **Comprehensive Identification of RNA-Binding Domains in Human Cells.** *Mol. Cell* 2016, **63**:696–710.

3. Morgan M: *AnnotationHub: Client to access AnnotationHub resources*. 2017.

4. Forbes SA, Beare D, Gunasekaran P, Leung K, Bindal N, Boutselakis H, Ding M, Bamford S, Cole C, Ward S, et al.: **COSMIC: exploring the world's knowledge of somatic mutations in human cancer.** *Nucleic Acids Res.* 2015, **43**:D805–11.

5. Amberger J, Bocchini C, Hamosh A: **A new face and new challenges for Online Mendelian Inheritance in Man (OMIM®).** *Hum. Mutat.* 2011, **32**:564–567.

6. Wickham H: *httr: Tools for Working with URLs and HTTP*. 2017.

7. Wickham H, Hester J, Ooms J: *xml2: Parse XML*. 2017.

8. Gene Ontology Consortium: **Gene Ontology Consortium: going forward.** *Nucleic Acids Res.* 2015, **43**:D1049–56.

9. Carlson M: *GO.db: A set of annotation maps describing the entire Gene Ontology*. 2017.

10. Finn RD, Attwood TK, Babbitt PC, Bateman A, Bork P, Bridge AJ, Chang H-Y, Dosztányi Z, El-Gebali S, Fraser M, et al.: **InterPro in 2017-beyond protein family and domain annotations.** *Nucleic Acids Res.* 2017, **45**:D190–D199.

11. Szklarczyk D, Franceschini A, Wyder S, Forslund K, Heller D, Huerta-Cepas J, Simonovic M, Roth A, Santos A, Tsafou KP, et al.: **STRING v10: protein-protein interaction networks, integrated over the tree of life.** *Nucleic Acids Res.* 2015, **43**:D447–52.

12. Bostock M, Rodden K, Russell K: *sunburstR: 'Htmlwidget' for 'Kerry Rodden' “d3.js” Sequence Sunburst*. 2017.

13. Fellows I: *wordcloud: Word Clouds*. 2014.

14. The UniProt Consortium: **UniProt: the universal protein knowledgebase.** *Nucleic Acids Res.* 2017, **45**:D158–D169.

15. Finn RD, Coggill P, Eberhardt RY, Eddy SR, Mistry J, Mitchell AL, Potter SC, Punta M, Qureshi M, Sangrador-Vegas A, et al.: **The Pfam protein families database: towards a more sustainable future.** *Nucleic Acids Res.* 2016, **44**:D279–85.

16. Dosztányi Z, Csizmók V, Tompa P, Simon I: **The pairwise energy content estimated from amino acid composition discriminates between folded and intrinsically unstructured proteins.** 2005, **347**:827–839.

17. Zhang J, Baran J, Cros A, Guberman JM, Haider S, Hsu J, Liang Y, Rivkin E, Wang J, Whitty B, et al.: **International Cancer Genome Consortium Data Portal--a one-stop shop for cancer genomics data.** *Database (Oxford)* 2011, **2011**:bar026.

18. Ooms J: **The jsonlite Package: A Practical and Consistent Mapping Between JSON Data and R Objects**. *arXiv:1403.2805 [stat.CO]* 2014, [no volume].

19. Ou J, Wang Y-X, Zhu LJ: *trackViewer: A bioconductor package with minimalist design for drawing elegant tracks or lollipop plot*. 2017.
